# Spatial distribution of FoxP3+ and CD8+ tumour infiltrating T cells reflects their functional activity

**DOI:** 10.18632/oncotarget.11039

**Published:** 2016-08-03

**Authors:** Rebecca Posselt, Katharina Erlenbach-Wünsch, Matthias Haas, Jonas JeΔberger, Maike Büttner-Herold, Marlen Haderlein, Markus Hecht, Arndt Hartmann, Rainer Fietkau, Luitpold V. Distel

**Affiliations:** ^1^ Department of Radiation Oncology, Universitätsklinikum Erlangen, Friedrich-Alexander-Universität Erlangen-Nürnberg, Erlangen, Germany; ^2^ Institute of Pathology, Universitätsklinikum Erlangen, Friedrich-Alexander-Universität Erlangen-Nürnberg, Erlangen, Germany; ^3^ Department of Radiology, Charité Universitätsmedizin, Berlin, Germany; ^4^ Deparment of Nephropathology, Institute of Pathology, Universitätsklinikum Erlangen, Friedrich-Alexander-Universität Erlangen-Nürnberg, Erlangen, Germany

**Keywords:** regulatory T cells, cytotoxic T cells, FoxP3+, CD8+, tumour-infiltrating lymphocytes

## Abstract

**Background:**

Regulatory and cytotoxic T cells are key players in the host's anticancer immune response. We studied the spatial distribution of FoxP+ and CD8+ cells to identify potential interactions.

**Methods:**

In 202 patients 103 pre-radiochemotherapy biopsies and 153 post-radiochemotherapy tumour specimens of advanced rectal cancer were available and an immunohistochemical double staining of FoxP3+ and CD8+ tumour-infiltrating lymphocytes was performed to investigate cell density and cell-to-cell distances.

**Results:**

FoxP3+ cells decreased after radiochemotherapy by a factor of 3 while CD8+ cells remained nearly unchanged. High epithelial (p=0.033) and stromal (p=0.009) FoxP3+ cell density was associated with an improved overall survival. Cell-to-cell distances of randomly distributed cells were simulated and compared to observed cell-to-cell distances. Observed distances shorter than the simulated, random distances were hypothesized to represent FoxP3+ cells actively interacting with CD8+ cells. Epithelial short distances were associated with a favourable prognosis while the opposite was true for the stromal compartment.

**Conclusion:**

The analysis of cell-to-cell distances may offer a tool to predict outcome, maybe by identifying functionally active, interacting infiltrating inflammatory cells in different tumour compartments.

## INTRODUCTION

In recent years the significance of inflammatory cells in cancer therapy has become a central interest of research. It is assumed that tumour infiltrating inflammatory cells (TIC) influence the patients' prognoses as key players in the intratumoural immune microenvironment. TIC are of paramount importance when it comes to so-called immunomodulatory effects of cancer therapy. It has been shown that in addition to their cytotoxic effects chemotherapeutic and radiotherapeutic treatment has an immune-priming effect in the tumour microenvironment [1, 2]. Antigen presenting cells initiate and shape the immune response, cytotoxic cells kill cancer cells and regulatory cells modulate the immune system and can impair the anti-tumoural immune response. The concept of immunosurveillance proposes that cancer cells can only survive when they either escape immune recognition or generate an immunosuppressive environment [3]. The induction of immunosuppressive cells is a major immune escape mechanism [4]. Tregs can suppress immune response by both cytokines and cell-to-cell contact [5]; they downregulate activation, proliferation and effector functions in CD4+ T cells, CD8+ CTL, natural killer and natural killer T cells, B cells and antigen-presenting cells [6]. Tregs are generally suspected to correlate with poor prognosis, even though there are also reports associating Tregs with a beneficial prognosis [7]. The presence of CD8+ CTL is widely associated with a favourable prognosis [4, 7]. Tregs and CTL are therefore counteractors promoting tumour escape and immunosurveillance, respectively.

Therefore, we focused on CD8+ CTL and FoxP3+ cells spatial distribution in advanced rectal cancer patients. We studied changes induced by radiochemotherapy (RCT) in tissue samples taken prior to and after RCT. We were especially interested in spatial CD8+ to FoxP3+ cell interrelations [8]. So in addition to the density of TIC in tumour tissue, the topographic relationship was calculated. Thus, the relevance of CD8+ to FoxP3+ distances, as a potential marker of their functional interaction, was assessed.

## RESULTS

### Pre- and post-RCT tissue samples of rectal cancer patients

**Figure 1:**
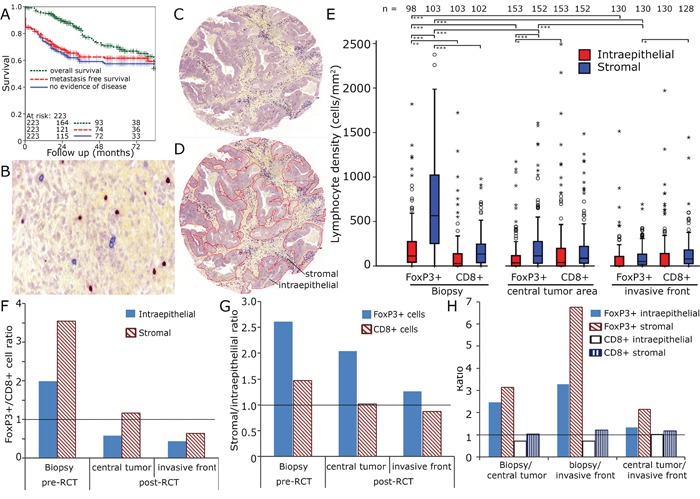
CD8+ and FoxP3+ cell densities in rectal adenocarcinoma Kaplan Meier plots for overall survival, metastasis free survival and no evidence of disease in the complete cohort **A.** Enlarged section (1:200) of tumour stroma with immunohistochemical double staining for FoxP3 (red nucleic staining) and CD8 (blue predominantly membranous staining) **B.** Tissue samples were processed into tissue microarrays using a core diameter of 2 mm **C.** Epithelial and stromal compartments were separately analysed **D.** Lymphocyte densities (cells/mm^2^) in the biopsy, the tumour centre and the invasive front **E.** FoxP3+/CD8+ ratio in stromal and epithelial compartment **F.** Stromal/epithelial ratio of FoxP3+ and CD8+ cells **G.** Ratio of pre-RCT biopsies, central tumour and invasive front of epithelial and stromal FoxP3+ and CD8+ cells **H.**

Clinical characteristics of the study group are given in Table 1. The median follow-up was 3.6 years. In the group with pre-RCT biopsies tissue samples from 103 patients and in the post-RCT group samples from 153 patients were available. Both biopsies and cancer resections were available from 54 patients. In both groups T3 tumours were most frequent (77.7% in the pre-RCT and 73.2% in the post-RCT group) with frequent lymph nodes metastases (68.9%/65.4%) and distant metastasis (15.5%/15.7%). 5-FU+Oxaliplatin (44.7%/57.5%) were the most frequently used chemotherapeutics followed by 5-FU alone (38.8%/31.4%). Overall-, metastasis-free survival (MFS) and no evidence of disease (NED-) survival rates of all patients were 66.8%, 61.9% and 57.4% at 6 years, respectively (Figure 1A). In the subgroup with biopsy samples prior to radiochemotherapy OS, MFS and NED-survival rates were 64%, 60.7% and 55.9%, respectively, for the complete cohort. In the subgroup with tumour samples after radiochemotherapy OS, MFS and NED-survival rates were 62.4%, 61.8% and 59.2% at 6 years.

**Table 1:** Clinical characteristics of 202 rectal cancer patients

103 patients with available biopsies	
Gender:	male: 63 (61.2%) female: 40 (38.8%)
Age:	<63: 53 (51.5%) >63: 50 (48.5%)
Primary tumour:	T2: 16 (15.5%) T3: 80 (77.7%) T4: 7 (6.8%)
Regional lymph nodes:	N0: 32 (31.1%) N1: 71 (68.9%)
Distant metastasis:	M0: 87 (84.5%) M1: 16 (15.5%)
UICC disease stage:	UICC stage I: 6 (5.8%) II: 25 (24.3%) III: 56 (54.4%) IV: 16 (15.5%)
Grading:	grade 1: 4 (3.9%) grade 2: 83 (80.6%) grade 3: 16 (15.5%)
Chemotherapeutic treatment:	none: 5 (4.9%) 5-FU: 40 (38.8%) 5-Fu+Oxaliplatin: 46 (44.7%) others: 12 (11.7%)

153 patients with available tumour resections	
Gender:	male: 118 (77.1%) female: 35 (22.9%)
Age:	<63: 71 (46.4%) >63: 82 (53.6%)
Primary tumour:	T2: 22 (14.4%) T3: 112 (73.2%) T4: 19 (12.4%)
Regional lymph nodes:	N0: 53 (34.6%) N1: 100 (65.4%)
Distant metastasis:	M0: 129 (84.3%) M1: 24 (15.7%)
UICC disease stage:	UICC stage I: 9 (5.9%) II: 38 (24.8%) III: 82 (53.6%) IV: 24 (15.7%)
Grading:	grade 1: 2 (1.3%) grade 2: 121 (79.1%) grade 3: 30 (19.6%)
Chemotherapeutic treatment:	none: 3 (2%) 5-FU: 48 (31.4%) 5-Fu+Oxaliplatin: 88 (57.5%) others: 14 (9.2%)

### CD8+ and FoxP3+ cell counts in pre- and post-RCT rectal cancer tissue

We performed double stainings for CD8+ CTL and FoxP3+ cells (Figure 1B - 1D) in pre-RCT biopsies and in post-RCT tumour sections. Post-RCT central tumour region and the invasive front were analysed separately. Both inflammatory cell types were quantified in the tumour stromal and the epithelial compartment. Prior to RCT FoxP3+ cells were two times more frequent epithelially than CD8+ cells and 3.5 times more frequent in the stromal compartment (Figure 1E, 1F). In the stromal compartment distinctly more FoxP3+ cells were present compared to the epithelial compartment (Figure 1E, 1G). RCT lasted for 38 days and surgery was performed 56 days after the end of RCT. Prior to RCT especially high numbers of FoxP3+ cells (698 ± 578 cells mm^−2^) were observed in the stromal compartment and after surgery FoxP3+ cell counts had clearly declined (central tumour 222 ± 306 cells mm^−2^, invasive front 103 ± 131 cells mm^−2^) to levels similar to or lower than CD8+ cell counts (Figure 1E, 1H). Given the enormous change of the FoxP3+ cell counts after RCT we were interested in the relationship of the cell counts prior to and after RCT. Therefore, we correlated cell counts and found strong correlations between epithelial and stromal CD8+ and FoP3+ cell counts and in central tumour and invasive front between stromal and epithelial CD8+ counts. However, we did not find a correlation between cell counts pre- and post-RCT (Supplementary Figure 1, supplementary Table 1).

### CD8+ and FoxP3+ densities influence prognosis

**Figure 2:**
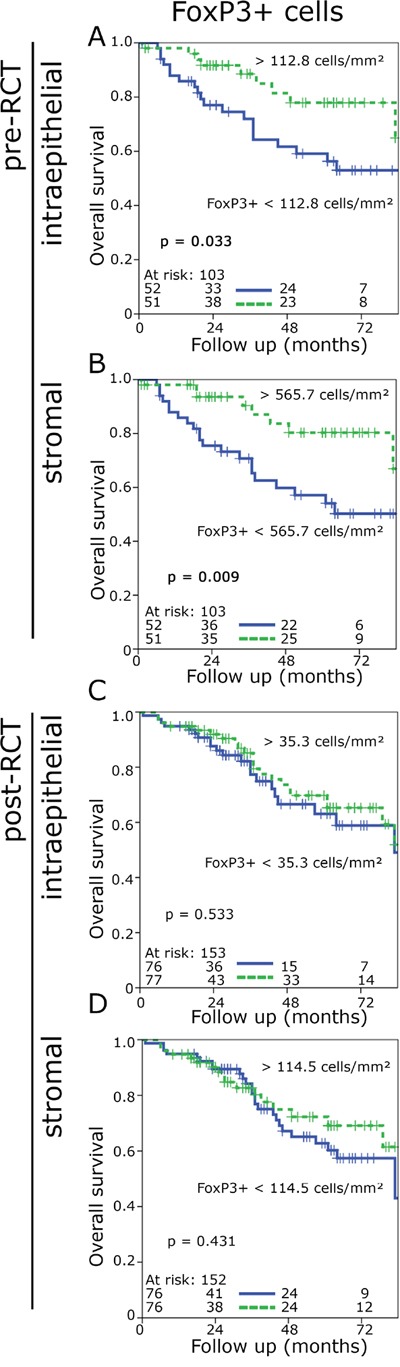
Kaplan-Meier plots for the densities of FoxP3+ cells in the stromal and epithelial compartment in pre-RCT biopsies and post-RCT central tumour (overall survival) FoxP3+cell density in epithelial pre-RCT biopsies **A.**, FoxP3+ cell density in the stromal pre-RCT biopsies **B.**, FoxP3+ cell density in the epithelial post-RCT central tumour **C.**, FoxP3+ cell density in the stroma post-RCT central tumour **D.**

Prior to RCT high FoxP3+ cell counts in both the epithelial (p = 0.033) and stromal compartment (p = 0.009) were associated with an improved OS and a tendency to improved NED-survival in the stromal compartment (p = 0.07) (Figure 2A, 2B, supplementary Figure 2A, C). In post-RCT samples the impact of Foxp3+ cells on prognosis was lost (Figure 2C, 2D, supplementary Figure 2E, G). CD8+ cells had no impact on OS before and after RCT (supplementary Figure 3A-D; supplementary Figure 2B, D, F, H). However, with regard of NED-survival high epithelial CD8+ counts showed a slight trend to a favourable prognosis (p = 0.099) and in the stromal compartment there was a distinct association with an improved prognosis (p = 0.001) (Supplementary Figure 2 F, H).

### CD8+ and FoxP3+ cell-to-cell distances

**Figure 3:**
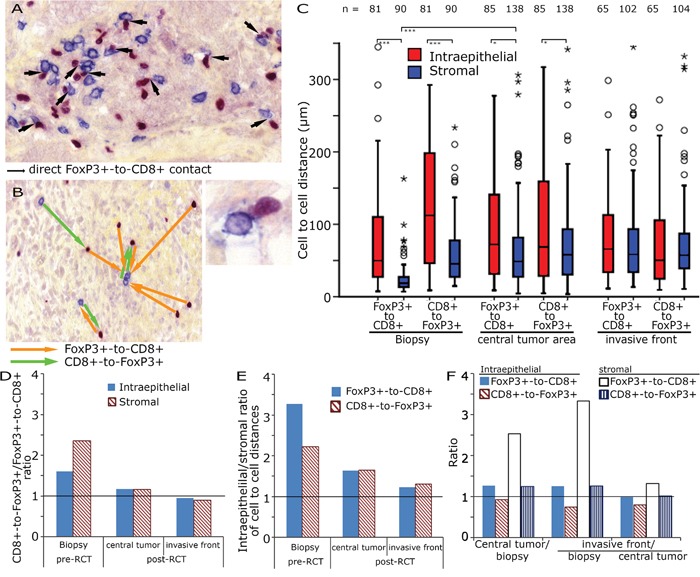
CD8+ to FoxP3+ cell distances in rectal adenocarcinoma Double staining for FoxP3+ and CD8+ in tumour stroma with highlighted direct cell-to-cell interactions (arrows, 200x original magnification) and a direct FoxP3+ to CD8+ interactions at higher magnification, bottom right **A.** Example for the calculation of the shortest distance between FoxP3+ and CD8+ cells (orange arrows) and CD8+ to FoxP3+ (green arrows) **B.** Cell-to-cell distances (μm) in the biopsy, the central tumour area and the invasive front **C.** CD8+-FoxP3+/FoxP3+-CD8+ ratio in preRCT biopsies and post RCT central tumour and invasive front **D.** Epithelial/stromal ratio of FoxP3+-CD8+ and CD8+-FoxP3+ distances **E.** Ratio of preRCT biopsies, central tumour and invasive front of epithelial and stromal FoxP3+-CD8+ and CD8+-FoxP3+ distances **F.**

After RCT FoxP3+ cell counts decreased dramatically and lost their prognostic significance. We hypothesized that cell-to-cell interactions were altered by a RCT induced modification of the tumoural microenvironment. Therefore, we studied the spatial distribution of the inflammatory cells (Figure 3A). The distance of each FoxP3+ cell and its nearest CD8+ cell (FoxP3+-CD8+) and the shortest distance between each CD8+ cell and the nearest FoxP3+ cell (CD8+-FoxP3+) were calculated (Figure 3B). The CD8+-FoxP3+ distances in pre-RCT biopsies were longer than the FoxP3+-CD8+ in both the epithelial and the stromal compartment. In post-RCT tissues this difference disappeared (Figure 3C, 3D). Epithelial cell-to-cell distances were longer than stromal cell-to-cell distances (Figure 3C, 3E) and stromal FoxP3+-CD8+ distances were longer in post-RCT tissues compared to pre-RCT biopsies (Figure 3C, 3F).

### CD8+ and FoxP3+ cell-to-cell distances influence prognosis

**Figure 4:**
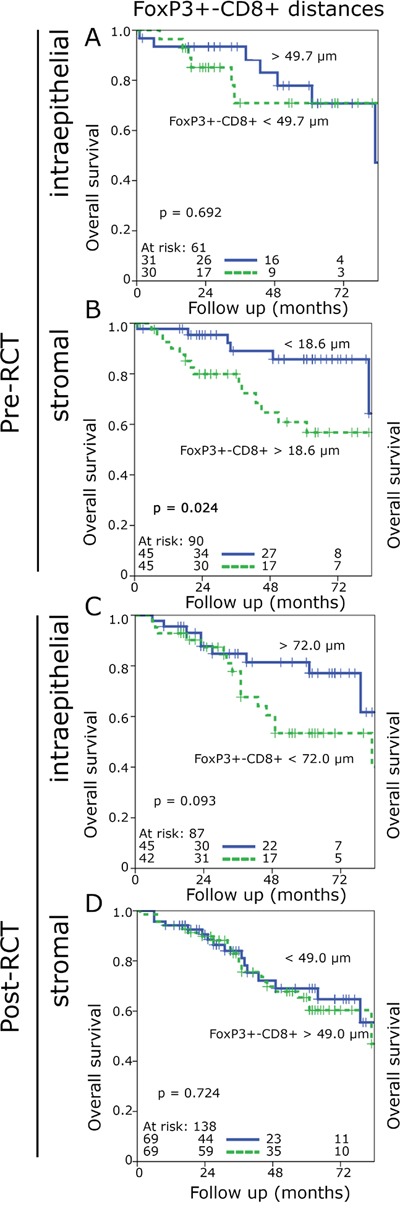
Kaplan-Meier plots for the cell-to-cell distances of FoxP3+-CD8+ distances in the stromal and epithelial compartment of preRCT biopsies and post-RCT central tumour specimens (overall survival) FoxP3+-CD8+ cell distances in the epithelial pre-RCT biopsies **A.**, FoxP3+-CD8+ cell distances in the stromal pre-RCT biopsies **B.**, FoxP3+-CD8+ cell distances in the epithelial post-RCT central tumour **C.**, FoxP3+-CD8+ cell distances in the stromal post-RCT central tumour **D.**

Short FoxP3+-CD8+ distances in the pre-RCT biopsies in the stroma were associated with improved OS, whereas no significant prognostic effect was observed for the epithelial compartment or with regard to NED-survival (Figure 4A, 4B, supplementary Figure 4 A, C). CD8+-FoxP3+ distances had no impact on OS or on NED prior to RCT (Supplementary Figure 4 B, D and 5 A, B). In contrast, short FoxP3+-CD8+ distances and short CD8+-FoxP3+ distances in the epithelial compartment post-RCT were associated with a clear trend to an inferior outcome (Figure 4C, supplementary Figure 4 E, F and 5 C). In the stromal compartment the distances had almost no influence on OS and only the CD8+-FoxP3+ distances an impact on NED (Figure 4D, supplementary Figure 4 G, H and 5 D).

### Comparison of observed to expected cell-to-cell distances

**Figure 5:**
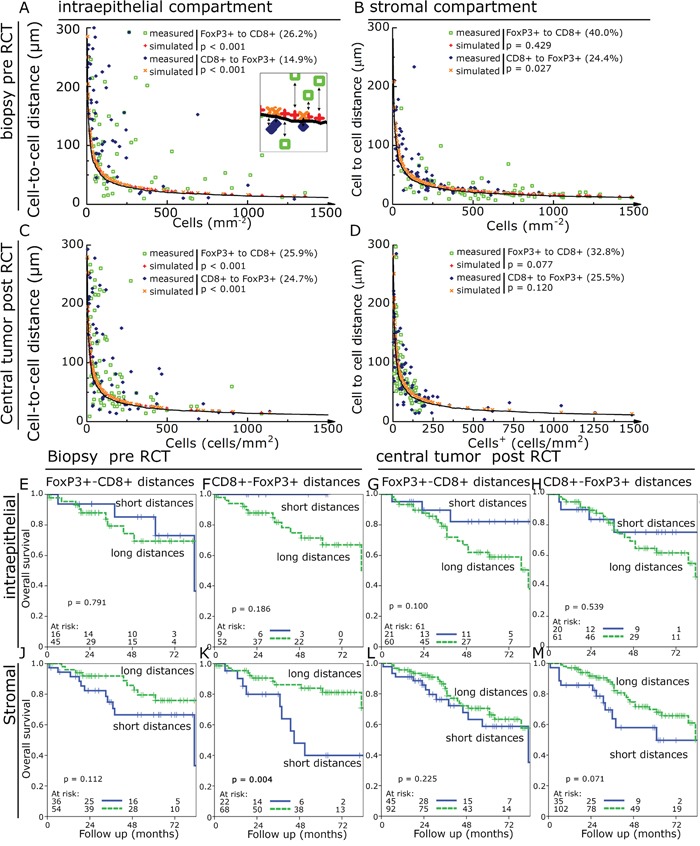
Observed compared to expected cell-to-cell distances Observed cell-to-cell distances in dependence of cell densities (green open squares and blue diamonds) were compared to simulated values (red plus and orange crosses). The solid line indicates 10% shorter distance than the simulated values and was used as cut off for defining observed distances shorter than the expected values. Cell-to-cell distances compared to cell densities in pre-RCT biopsies in the epithelial compartment **A.** and in the stromal compartment **B.** as well as in post-RCT central tumour in the epithelial **C.** and stromal compartment **D.** Kaplan-Meier plots using the above identified samples with shorter distances than expected compared to longer distances for OS. FoxP3+-CD8+ cell distances in the epithelial pre- RCT biopsies **E.**; CD8+-FoxP3+ cell distances in the epithelial pre-RCT biopsies **F.**; FoxP3+-CD8+ cell distances in the epithelial post-RCT central tumour **G.**; CD8+-FoxP3+ cell distances in the epithelial post-RCT central tumour **H.** and FoxP3+-CD8+ cell distances in the stromal pre-RCT biopsies **J.**; CD8+-FoxP3+ cell distances in the stromal pre-RCT biopsies **K.**; FoxP3+-CD8+ cell distances in the stromal post-RCT central tumour **L.**; CD8+-FoxP3+ cell distances in the stromal post-RCT central tumour **M.**

**Figure 6:**
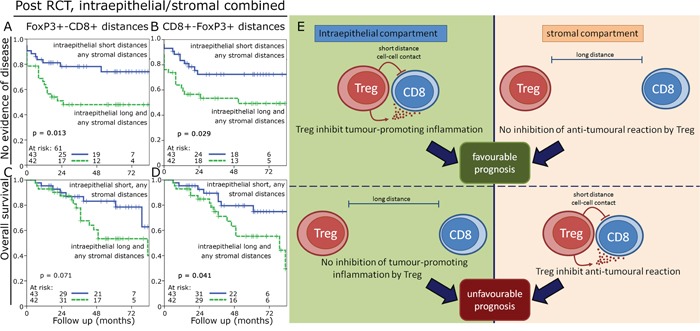
Kaplan-Meier plots for the combination of cell-to-cell distances Kaplan-Meier plots using the ratio of short epithelial distances with the respective stromal distances compared to long epithelial distances with the respective stromal distances. NED survival in the post-RCT samples for FoxP3+-CD8+ cell distances **A.** and CD8+-FoxP3+ cell distances **B.** and OS for FoxP3+-CD8+ cell distances **C.** and CD8+-FoxP3+ cell distances **D.** Model for epithelial and stromal cell-to-cell interactions and their prognostic values **E.**

One challenge of using cell-to-cell distances is that in tissues with high cell densities a priori shorter distances are found compared to tissues with lower cell densities. We hypothesized that dysfunctional FoxP3+ cells would be randomly distributed in the tissue. In contrast we would expect that functional FoxP3+ cells interacting with CD8+ cells would be in greater proximity to each other than expected for randomly distributed cells. So we simulated for each cell density an expected cell-to-cell distance of randomly distributed cells and compared it to the measured cell-to-cell distance (Figure 5A – 5D). Observed cell-to-cell distances which were at least 10% shorter than the expected distance were rated as “short distance” and were compared to the longer distances. Between 14.9% and 40.0% of the investigated specimens were found to have short cell-to-cell distances and the FoxP3+ cells in these tissues were regarded as being functional. Observed cell-to-cell distances in the stromal compartment were much closer to the expected random values (p > 0.02) than in the epithelial compartment (p < 0.001). Using this approach we found that in the epithelial compartment pre- and post-RCT short cell-to-cell distances had a trend to be associated with an improved OS (figure 5E-5H). In the stromal compartment this effect reversed and short distances were associated with a trend towards an unfavourable OS reaching significance for short CD8+-FoxP3+ distances in pre-RCT tissue (figure 5J-5M). Post-RCT a ratio of short epithelial distances with the respective stroma distances of the same case could predict a favourable NED and OS compared to the ratio of long epithelial distances divided by the respective value in the stroma (figure 6A-6D).

## DISCUSSION

We used a double staining approach of CD8+ and FoxP3+ cells including whole slide scanning of tissue microarrays and advanced image analysis software. The software enables to count both cell types and identifies for each positive cell the closest cell of the other cell type. In our neoadjuvantly treated rectal cancer cohort we found high numbers of FoxP3+ cells especially in the stromal compartment of pre-RCT biopsies. This is in line with previous reports [9]. We observed an extraordinarily strong decrease of FoxP3+ cell densities after RCT while CD8+ counts were hardly influenced. As the tumour resection was performed 8 weeks after RCT, we hypothesized that the decrease of FoxP3+ inflammatory cells was rather a change of the tumour microenvironment induced by RCT than a direct cytotoxic effect of the therapy. This idea is supported by the fact that FoxP3+ cells are usually assumed to be radioresistant cells [10, 11]. In early reports high numbers of FoxP3+ cells in cancer were associated with an unfavourable prognosis [12] potentially by inhibiting an effective anti-tumoural immunoreaction. RCT might therefore promote an improved immunological profile with reduced numbers of FoxP3+ Treg and stable numbers of cytotoxic T cells under the assumption that FoxP3+ cells induce an immunologic tolerance [13]. In contrast to this model, however, in the present study high FoxP3+ cell densities in the epithelial and stromal compartment were associated with a favourable outcome. Recently Treg were associated with good cancer specific survival [14, 15] and overall survival [16] in colorectal cancer. One meta-analysis of six studies evaluating the impact of Treg infiltration in colorectal cancer could not show an association of Treg and survival [17]. Here we found a clear association of FoxP3+ cells and improved OS. A reason may be that our cohort includes only rectal cancer cases consisting of a homogenous group of cancer patients with a uniform stage of disease and treatment.

We were particularly interested in the spatial distribution of FoxP3+ cells+ and CD8+ cells and their cell-to-cell interactions [18]. Our fundamental hypothesis was that short distances between FoxP3+ cells and CTL indicate functional cells, while long distances designate dysfunctional cells [19]. However, the use of distances without keeping the factor of cell density in mind is only of limited value, because inevitably high densities of infiltrating cells in a tissue are associated with shorter intercellular distances. Therefore we compared observed cell densities of each investigated sample with mathematically simulated cell-to-cell distances of “randomly” distributed cells. Samples with shorter cell-to-cell distances than the simulated distances were rated as probably functional cells having FoxP3+-CD8+ cell interactions. Accordingly, we could show that short intercellular distances in the epithelial compartment showed a trend towards a favourable prognosis, whereas in the stromal compartment long distances predicted an improved prognosis. According to our theory this would indicate that functional inflammatory cells in the tumour epithelium convey a favourable course of disease and vice versa in the stromal compartment dysfunctional cells are favourable for prognosis. Close CD8+-FoxP3+ interactions may be favourable due to a down-regulated inflammatory environment, which can promote tumour growth [20–22] (Figure 6E). We observed that in the epithelial compartment 15% to 25% of the samples had presumably functional cells and in the stromal compartment in 24% to 40% of the tissues functional cells were observed. This may be explained by the fact that in the epithelial compartment a immunosuppressive environment is induced by the cancer cells themselves, for example by expressing PD-L1 or other suppressing factors. In the non-neoplastic stromal compartment the suppression of inflammatory cells via direct cell contact to tumour cells is lacking. These observations implicate that within one single tissue the same cell type can have either a positive or negative prognostic impact depending on the microenvironment in which it is residing. We showed previously that in gastric cancer short CD8+-FoxP3+ distances both in the epithelial and stromal compartment are associated with a favourable metastasis-free survival and OS [23]. In an anal cancer cohort we studied the spatial distribution of dendritic cells (CD1a+), B cells (CD20) and Treg (FoxP3+) and found most of the dendritic cells and epithelial B cells to be non-functional, while stromal B cells and FoxP3+ cells were presumed to be functional cells [24].

Our fundamental thesis is that the distances between FoxP3+ cells and CD8+ lymphocytes are markers of their functional status. It is well accepted that Treg can suppress T cells by direct cell-cell contact or by the contact-independent mechanisms through the production of TGF-β, IL-10 and other immunosuppressive factors [25–28]. Short distances are the pre-condition for cell-to-cell contact mechanisms. For intercellular communication via soluble factors or exosomes the distance is less significant. However, due to the gradient of soluble factors one can assume that the effect of such factors is greatest on closely located cells. Thus shorter distances could be indicative of a higher probability of cell-to-cell contact mechanisms as well as mechanisms mediated by soluble factors. Thus we assume that analysis of distances can identify both FoxP3+ cells acting via cell-to-cell contact and those that have the greatest impact on neighbouring cells through soluble factors. Another constraint is that there could be other cells expressing FoxP3+ besides regulatory T cells and regulatory T cells which lack FoxP3 expression [29]. Yet, in the absence of alternative Treg markers FoxP3+ is considered to be the most specific Treg marker in paraffin embedded tissues so far [28, 30]. Our analysis refers to the FoxP3+ cells for we consider that most of these cells are Treg.

We used only two TMA tissue spots of each patient's pre-RCT biopsies and for each of the two post-RCT group samples. Therefore, we cannot exclude that due to heterogeneity in the tumour infiltrate sampling error might influence the comparability of the probes. In an earlier study analysing B-cell infiltrate in classical Hodgkin lymphoma we could, however, show a positive correlation between results in whole block sections and a single TMA core [31]. In line with this, in a study of gastric adenocarcinoma of the cardia we compared the tumour infiltrating cell counts in TMA spots with whole block staining and found similar results [32]. Moreover, even the use of whole block sections cannot ensure that no sampling error takes place: with many tumours measuring several cm^2^ the tissue blocks prepared by the pathologist only represent part of the tumour and the evaluated sections have a thickness of only a few μm. To try to assess immunological differences within one tumour a specially designed study beyond the scope of the present investigation would be needed.

In summary FoxP3+ cells were associated with an improved prognosis in advanced rectal cancer. In the epithelial compartment short CD8+-FoxP3+ distances were prognostically favourable whereas in the stromal compartment a lack of interaction was beneficial. This might indicate that whilst an inhibitory effect of Treg on inflammation as a potential promotor of tumour progression might be favourable in the tumour epithelium, in the stroma a lack of FoxP3+ cell-CTL interaction might indicate a non-suppressed T-cell response to the cancer conveying a better outcome. The analysis of cell-to-cell interactions may offer the possibility to discriminate functionally active from dysfunctional cells in tissue specimens.

## MATERIALS AND METHODS

### Patient selection

The patient cohort included 202 patients with advanced rectal cancer treated at the University Hospital of Erlangen between 2006 and 2013. All patients received a neoadjuvant RCT followed by total mesorectal excision (TME) -surgery. In 103 out of the 202 patients biopsies could be gained and in 153 patients resection samples were available. In 54 patients both a biopsy and resected tissue were available. The tumour staging was collected from the patients' files and performed according to the criteria of the International Union Against Cancer (UICC 2009). The Erlangen-Nürnberg Tumour Centre Database provided clinical patient data (Table 1). The Ethics Review Committee of the University Hospital Erlangen approved the study including the use of resected tissue and patients' survival data.

### Treatment protocol

Neoadjuvantly all patients received a RCT including a conventional radiotherapy with single fractions of 1.8 Gy up to a total dose of 50.4 Gy and a 5-fluorouracil-based chemotherapy over a period of 38 days. The tumour response was assessed eight weeks after RCT by proctoscopy, computed tomography pelvic scans, endorectal ultrasound and clinically. Thereafter, surgical cancer resection was performed in 192 patients. Ten patients had achieved a complete remission and surgery was not necessary. These 10 patients then received an adjuvant chemotherapy with 5-FU. Subsequently, patients received regular follow-ups.

### Tissue microarray and immunohistochemistry

Paraffin-embedded samples of 103 pre-RCT biopsies and 153 post-RCT tumour resections were processed into tissue microarrays (TMA) with a core diameter of 2mm. For this purpose two TMA spots of the biopsy, the invasive front and the tumour centre each per patient were inserted in a paraffin block.

An immunohistochemical double staining with FoxP3 and CD8-specific antibodies was performed. For antigen retrieval slides were cooked for 5 minutes in citrate buffer after deparaffination [33, 34]. After overnight incubation with a FoxP3-specific antibody in a 1:100 detection was performed with the Polymer-Kit (Fa. Zytomed POLAP-100) and Fast Red. In a second step a CD8-specific antibody was added in a 1:50 dilution with a 60 minutes reaction time, followed by detection with the Polymer-Kit and Fast Blue [35].

### Quantification of TILs

Stained slides were scanned with a high throughput scanner (Zeiss, Mirax MIDI Scan, Göttingen, Germany) at a magnification of 1:200 and transferred to a PC (1). TIC were counted with image processing software (Biomas, Erlangen, Germany). The epithelial and stromal compartment were marked each as separate areas. The size of the area (in mm^2^) was calculated automatically, the identification of the FoxP3+ and CD8+ cells took place semiautomatically by the software to determine the densities of the TIC.

### Cell-to-cell-distances

Mean cell-to-cell distances, which are influenced by cell density, were measured in the epithelial and stromal compartment separately, assessing distances of FoxP3+ to FoxP3+, CD8+ to CD8+, FoxP3+ to CD8+ and CD8+ to FoxP3+. Numbers of positive cells were set in relation to the surface area, and the analysis software calculated the mean distance values between the different cell types in each compartment. The equations for the more precise calculation of the cell-cell distances have already been described previously [23].

### Simulation of cell-to-cell distances of randomly distributed cells

Cell densities (cells/mm^2^) of FoxP3+ and CD8+ cells in each patient's tissue were used to calculate the expected cell-to-cell distances of randomly distributed cells. Random positioning of the cells was simulated with the aid of random numbers generated by visual basic software of the spreadsheet program Excel. The x and y coordinates of the quantity of both “cell types” were generated and the shortest distance of each cell to the nearest cell of the other type was calculated and the shortest distances were averaged. This procedure was repeated 200 times and the mean distance was calculated bidirectionally, both from the FoxP3+ cells to the nearest CD8+ cells and from the CD8+ cells to the nearest FoxP3+ cells.

### Statistical methods

Statistical analyses were performed using SPSS version 21. The examined clinical variables were tumour specific survival, overall survival, metastasis-free survival, local failure-free survival and no evidence of disease survival. They were calculated according to the Kaplan-Meier method and compared by the log-rank test. The cut-off value for the definition of subgroups was the median. Paired t-test was used to compare observed cell-to-cell distances with expected cell-to-cell distances.

## SUPPLEMENTARY FIGURES AND TABLE


