# Supplementary material for: Spatial distribution of FoxP3+ and CD8+ tumour infiltrating T cells reflects their functional activity
Source: Oncotarget. 2016 Aug 3;7(37):60383–94. doi: 10.18632/oncotarget.11039 (PMC5312390; doi:10.18632/oncotarget.11039)
Supplement: Supplementary file 1 [file oncotarget-07-60383-s001.pdf]

Spatial distribution of FoxP3+ and CD8+ tumour infiltrating T cells reflects their functional activity

SUPPLEMENTARY FIGURES AND TABLES

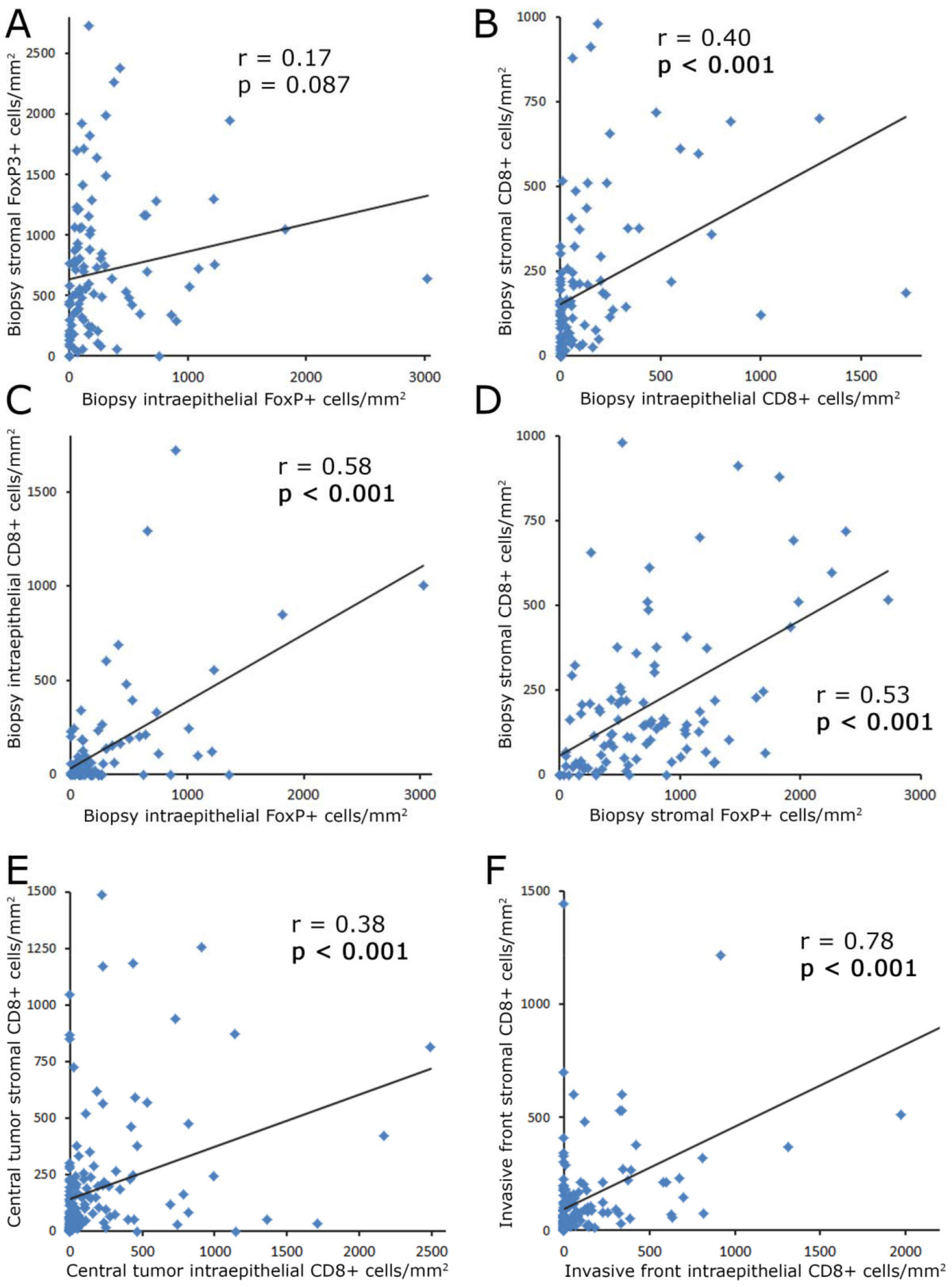

Supplementary Figure S1: Correlation of cell densities in the stromal and epithelial compartment. Cell densities in pre-RCT biopsies: Epithelial FoxP3+ cells compared to stromal FoxP3+ cells **A.**; epithelial CD8+ cells compared to stromal CD8+ cells **B.**; epithelial FoxP3+ cells compared to epithelial CD8+ cells **C.**; stromal FoxP3+ cells compared to stromal CD8+ cells **D.** Cell densities in post-RCT tumour resections: epithelial CD8+ cells compared to stromal CD8+ cells in the central tumour region **E.** and in the invasive front **F.**

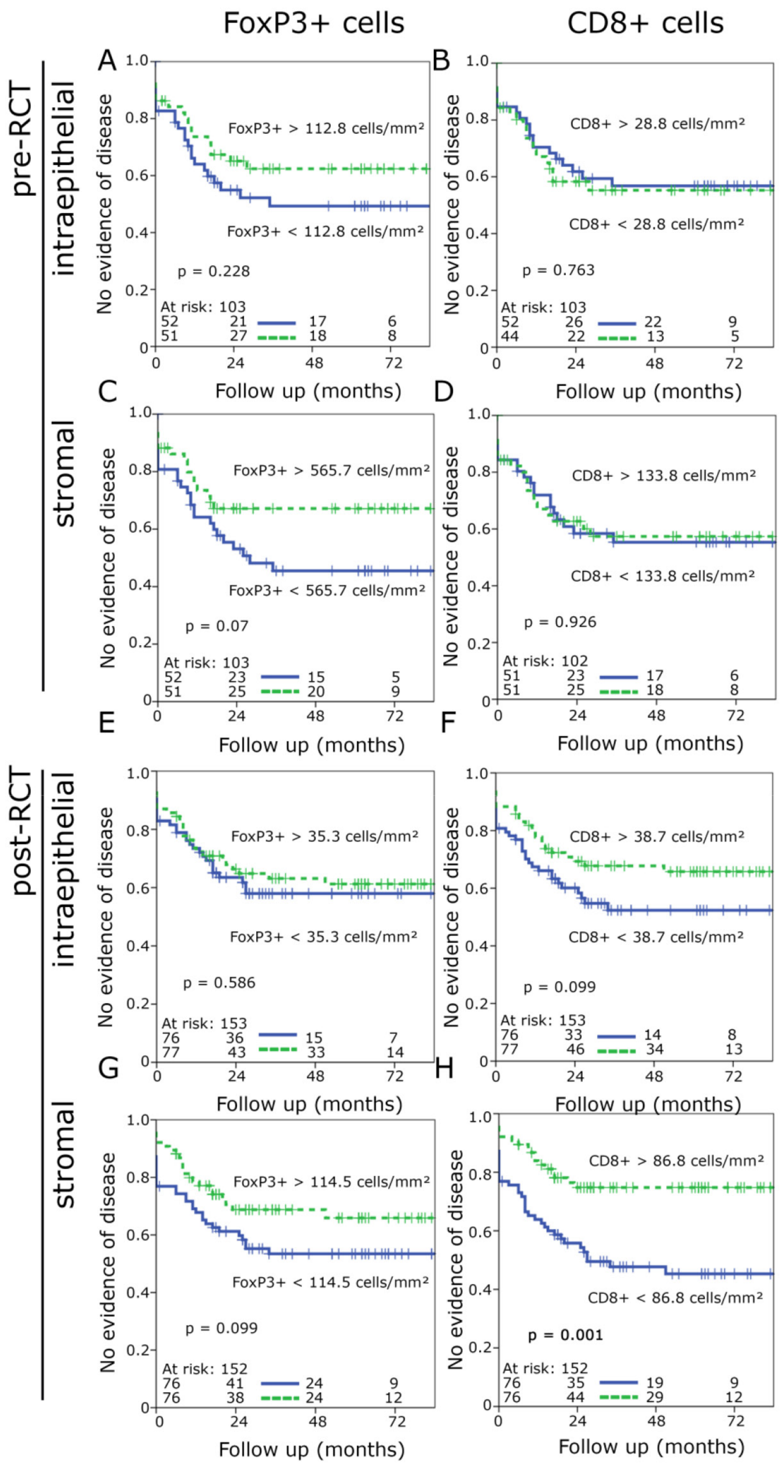

Supplementary Figure S2: Kaplan-Meier plots for the densities of FoxP3+ and CD8+ cells in the stromal and epithelial compartment of pre RCT biopsies and in post RCT central tumour (no evidence of disease survival). FoxP3+ density epithelial pre RCT biopsies **A.** CD8+ density epithelial pre RCT biopsies **B.** FoxP3+ density stromal pre RCT biopsies **C.** CD8+ density stromal pre RCT biopsies **D.** FoxP3+ density epithelial post RCT central tumour **E.** CD8+ density epithelial post RCT central tumour **F.** FoxP3+ density stromal post RCT central tumour **G.** CD8+ density stromal post RCT central tumour **H.**

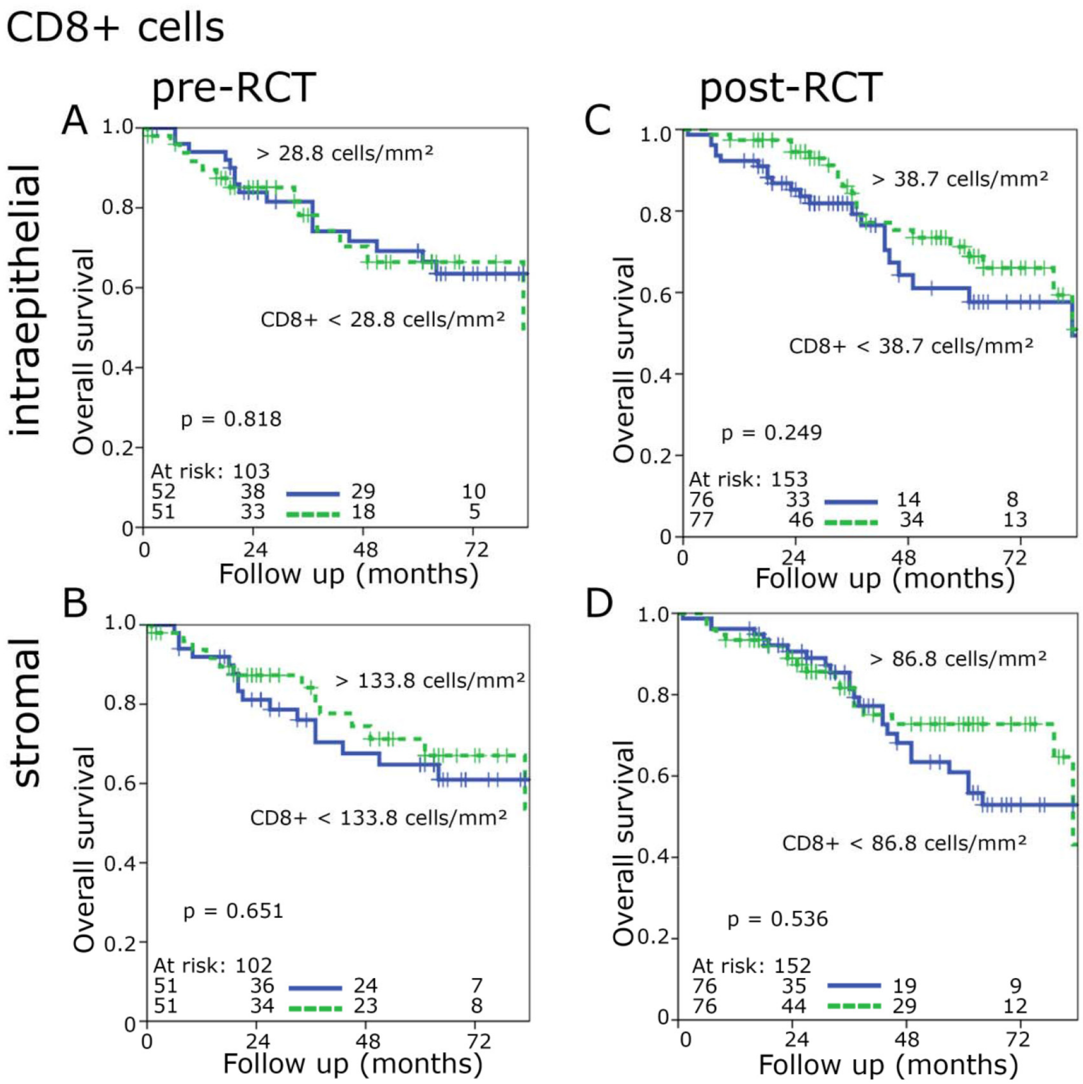

Supplementary Figure S3: Kaplan-Meier plots for the densities of CD8+ cells in the stromal and epithelial compartment of pre RCT biopsies and in post RCT central tumour (overall survival). CD8+ density epithelial pre RCT biopsies **A.**, CD8+ density stromal pre RCT biopsies **B.**, CD8+ density epithelial post RCT central tumour **C.**, CD8+ density stromal post RCT central tumour **D.**

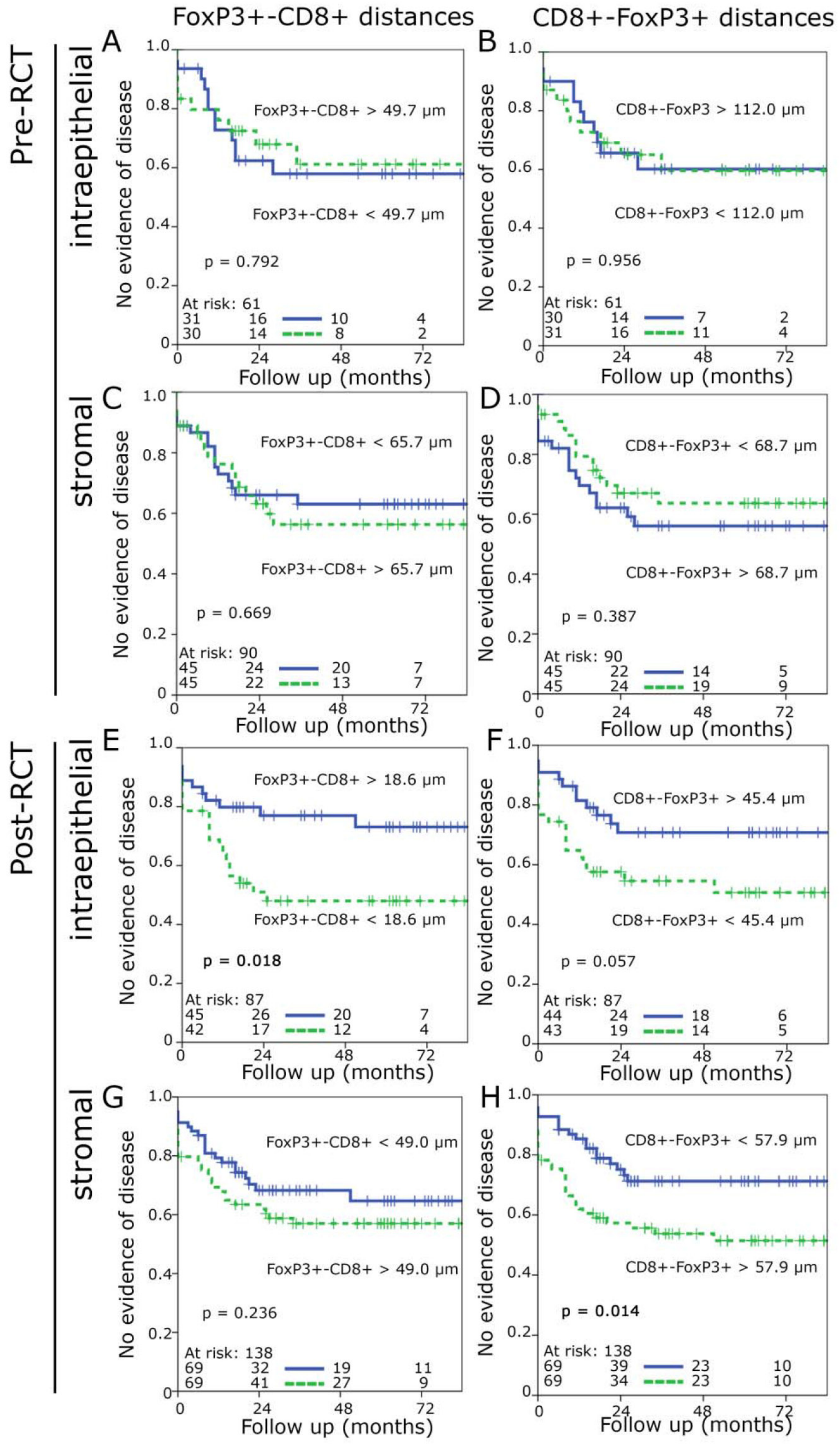

Supplementary Figure S4: Kaplan-Meier plots for the cell-cell distances of FoxP3+-CD8+ and CD8+-FoxP3+ cells in the stromal and epithelial compartment pre and post RCT in the central tumour (no evidence of disease survival). FoxP3+-CD8+ epithelial pre RCT biopsies **A.**, CD8+-FoxP3+ epithelial pre RCT biopsies **B.** FoxP3+-CD8+ stromal pre RCT biopsies **C.** CD8+-FoxP3+ stromal pre RCT biopsies **D.** FoxP3+-CD8+ epithelial post RCT central tumour **E.** CD8+-FoxP3+ epithelial post RCT central tumour **F.** FoxP3+-CD8+ stromal post RCT central tumour **G.** CD8+-FoxP3+ stromal post RCT central tumour **H.**

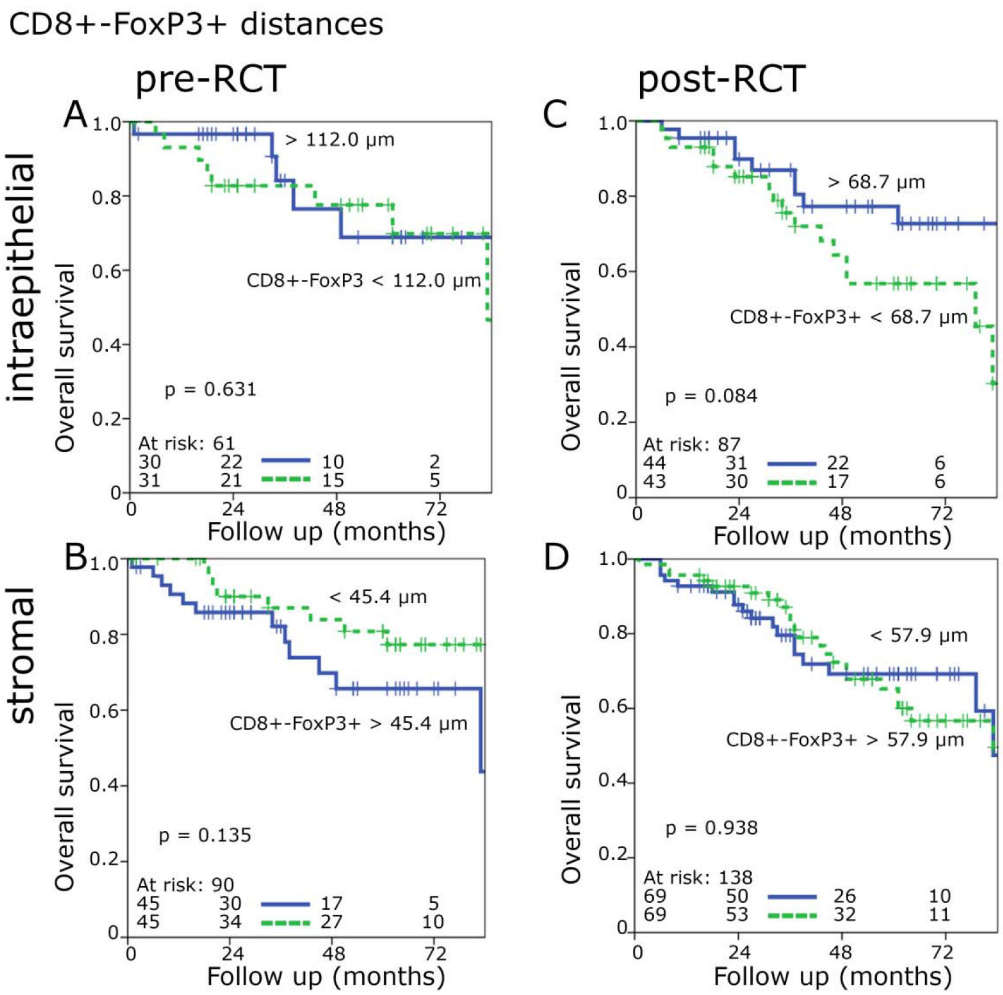

Supplementary Figure S5: Kaplan-Meier plots for the cell-cell distances of CD8+-FoxP3+ cells in the stromal and epithelial compartment pre and post RCT in the central tumour (overall survival). CD8+-FoxP3+ epithelial pre RCT biopsies **A.**, CD8+-FoxP3+ stromal pre RCT biopsies **B.**, CD8+-FoxP3+ epithelial post RCT central tumour **C.**, CD8+-FoxP3+ stromal post RCT central tumour **D.**

Supplementary Table S1: Correlations between CD8+ or FoxP3+ cells in different compartments, prior RCT (biopsy) or post RCT (central tumour, invasive front), respectively.

tissue	compartment	TIC		tissue	compartment	TIC	patients	r	p
biopsy	intraepithelial	FoxP3	-	biopsy	intraepithelial	CD8	103	0.582	< 0.001
biopsy	stromal	FoxP3	-	biopsy	stromal	CD8	103	0.529	< 0.001
biopsy	intraepithelial	CD8	-	biopsy	stromal	CD8	105	0.400	< 0.001
central tumour	intraepithelial	FoxP3	-	central tumour	intraepithelial	CD8	153	0.480	< 0.001
central tumour	intraepithelial	FoxP3	-	central tumour	stromal	CD8	152	0.481	< 0.001
central tumour	stromal	FoxP3	-	central tumour	stromal	CD8	152	0.281	< 0.001
central tumour	intraepithelial	CD8	-	central tumour	stromal	CD8	152	0.317	< 0.001
central tumour	stromal	CD8	-	invasive front	stromal	CD8	112	0.383	< 0.001
invasive front	intraepithelial	CD8	-	invasive front	stromal	CD8	128	0.776	< 0.001
